# Self-Crosslinkable Pressure-Sensitive Adhesives from Silicone-(Meth)acrylate Telomer Syrups

**DOI:** 10.3390/ma15248924

**Published:** 2022-12-14

**Authors:** Mateusz Weisbrodt, Agnieszka Kowalczyk

**Affiliations:** Department of Chemical Organic Technology and Polymeric Materials, Faculty of Chemical Technology and Engineering, West Pomeranian University of Technology in Szczecin, 70-322 Szczecin, Poland

**Keywords:** pressure-sensitive adhesives, telomerization, bulk photopolymerization, polyacrylates, silicone acrylates

## Abstract

In this study, a novel and environmentally friendly method for the preparation of photoreactive pressure-sensitive adhesives (PSAs) was demonstrated. Adhesive binders based on n-butyl acrylate, methyl methacrylate, acrylic acid, and 4-acryloyloxy benzophenone were prepared with a UV-induced telomerization process in the presence of triethylsilane (TES) as a telogen and acylphosphine oxide (APO) as a radical photoinitiator. The influence of TES (0–10 wt. parts) and APO (0.05–0.1 wt. parts/100 wt. parts of monomer mixtures) concentrations on the UV telomerization process kinetics was investigated using a photodifferential scanning calorimetry method and selected physicochemical features of the obtained silicone-(met)acrylate telomeric syrups (K-value, solid content, glass-transition temperature, and dynamic viscosity), as well as properties of the obtained PSAs (T_g_, adhesion, tack, and cohesion), were studied. An increase in TES content caused a significant decrease in the T_g_ values (approx. 10 °C) and K-value (up to approximately 25 a.u.) of the dry telomers, as well as the dynamic viscosity of the telomeric syrups. PSAs were obtained through UV irradiation of thin polymer films consisting only of silicone-(meth)acrylate telomer solutions (without the use of additional chemical modifiers or of a protective gas atmosphere and protective layers). PSAs were characterized by very good adhesion (12.4 N/25 mm), cohesion at 20 °C (>72 h) and 70 °C (>72 h), and low glass-transition temperature (−25 °C).

## 1. Introduction

Pressure-sensitive adhesives (PSAs) are viscoelastic materials that remain permanently adhesive and can adhere even under light pressure [1]. Among the many materials used in the preparation of PSAs, the most common are poly(acrylates); in particular, because of their excellent oxidation resistance, high transparency, high water resistance, and lack of yellowing. However, acrylic PSAs have disadvantages, such as low adhesion to low-energy substrates and low thermal stability [2,3,4,5]. Silicone PSAs do not have these disadvantages, but they are used as solvent-based systems (50 wt.%) associated with high emissions of volatile organic compounds (VOCs) during coating [6,7]. To obtain materials with the advantages of both while minimizing their disadvantages, acrylic adhesive binders can be modified with organosilicon compounds. For this purpose, polydimethylsiloxane (PDMS) is often used due to its significantly lower surface energy, non-toxicity, and non-flammable properties. Unfortunately, because of the low compatibility of these materials, creating a homogeneous mixture is difficult. Synthesis of silicone-acrylate resins is generally carried out by emulsion polymerization with the use of appropriate emulsifiers, but this results in the formation of a large amount of hard-to-clean wastewater [2,8,9]. Another approach is to obtain silicone-acrylic pressure-sensitive adhesives through polymerization of (meth)acrylate monomers using silicone macroinitiators. To increase the content of polydimethylsiloxane in the silicone-acrylic structure, the silicone chain is modified with urethane dimethacrylates. These structures can form a semi-interpenetrating polymer network. However, they are obtained by polymerization in an organic solvent, which results in high emission of VOCs in the drying channel, and the reaction products also have broad polydispersity (PDI > 6) [2,3].

To achieve high cohesion in PSAs, they are often crosslinked or formed into structures capable of creating physical interactions. The most popular physical interactions between polymer chains are van der Waals forces, those associated with a microphase separation, interactions between polar monomer units (e.g., acrylic acid, acrylamide), and ionic interactions. The bonds thus formed are non-permanent and can be reversed at the elevated temperature. Another way to increase the cohesion of a PSA is to disperse the filler in the adhesive binder. Unfortunately, this leads to an increase in viscosity and a decrease in adhesion and tack. Commonly used fillers are halloysite and silica [10]. In contrast, chemical crosslinking of adhesive binders can take place in many ways. Branched and then three-dimensional structures (gel formation), which are usually industrially undesirable, can already be formed in the reactor during the polymerization process when a high polymer content and a low monomer concentration are used. If desired, polyfunctional compounds (e.g., triacrylate of trimethylopropane) can be used in the polymerization reaction. The incorporation of epoxy groups, N-alkoxy amides, and organosilanes into the polymer chain enables the crosslinking of the chains with the use of appropriate catalysts. One-component systems (e.g., containing organometallic crosslinkers, metal salts, or hydrazines) and two-component systems (e.g., containing polyfunctional isocyanates, amino resins, polyfunctional azirdines, or peroxides), which enable crosslinking of the binder under the influence of various factors (i.e., temperature, humidity or time), have been studied [11,12]. Radiation-induced crosslinking is a dynamically developing field for the crosslinking of adhesives. In this way, the standard peroxides can be replaced by type I or type II photoinitiators. One special kind of photoinitiators are unsaturated (polymerizable) type II photoinitiators, which require a proton donor to form radicals. In the photocrosslinking processes involving their participation, no by-products are produced, which is of particular importance in the preparation of self-adhesive materials for medical purposes. Crosslinking of this type of photoinitiators is most often initiated with mercury lamps characterized by the emission of UV-C radiation with a length of 220–280 nm [11,13].

Telomerization may be a new, waste-free method of obtaining acrylic PSAs with embedded organosilicon particles. It is a chain reaction of a monomer (taxogen, M) with a reactive compound (telogen, YZ)—also called chain transfer agents (CTAs)—in which Y(M)_n_Z molecules (telomers/oligomers) are formed [14,15]. Telogens can be divided into three groups. The first group consists of halo compounds, the second of organic compounds containing an active center bound to the carbon atom (i.e., alcohols, carboxylic acids, amines, etc.), and the third group consists of compounds containing S-H, Si-H, or P-H bonds (sulfur, silicon, and phosphorus compounds) [16]. The telomerization process can be initiated by many factors; e.g., thermal initiators (organic peroxides, hydroperoxides, azo compounds, etc.), UV radiation, γ radiation accompanying beta decay of ^60^Co to nonradioactive ^60^Ni, and redox processes involving metal ions with variable valences [17]. However, thermally initiated reactions have the greatest importance (the process takes place at an elevated temperature of 60 ÷ 90 °C over several hours), with 2,2′-azobisisobutyronitrile being most often used for initiators [18,19,20,21,22,23,24]. There are also reports of UV-induced telomerization, which is the main research interest of the authors of the present article [25,26,27,28]. The telomerization process should be distinguished from controlled polymerization processes (e.g., ATRP, CRP), although the common feature is the presence of CTAs. What distinguishes controlled polymerization from telomerization is the CTA content (below 3 wt.% in the case of controlled polymerization and from 3 to 50 wt.% in telomer systems [29]) and the nature of the resulting macromolecules (in the case of polymerization, they are polymers and, less frequently, oligomers; in the case of telomerization, they are oligomers with characteristic end groups derived from telogen). As emphasized by the leading researchers studying the telomerization process, telomerization is not a living polymerization (such as ATRP or RAFT) but a partially controlled reaction to obtain macromolecules. The advantage of telomerization over controlled polymerization processes is its high efficiency and the ease of carrying out the process at an industrial scale (lower requirements for the purity of the reagents) [17].

This work describes a new, environmentally friendly method of obtaining pressure-sensitive adhesives with very good adhesive properties that are capable of self-crosslinking under the influence of UV radiation. The UV telomerization of (met)acrylate monomers with triethylsilane as the telogen in the presence of acylphosphine oxide as the radical photoinitiator was performed and is described in detail. Moreover, the influence of telogen concentration on the properties of the obtained silicone-(met)acrylate telomer syrups and PSAs in comparison with the materials obtained through photopolymerization was investigated.

## 2. Materials and Methods

### 2.1. Materials

For the preparation of silicone-(met)acrylate telomers (Si telomers), the following components were employed: n-buthyl acrylate (BA), acrylic acid (AA), and methyl methacrylate (MMA; BASF; Ludwigshafen, Germany) were used as monomers; 4-acryloylooxybenzophenone (ABP, Chemitec, Scandiccy, Italy) was used as the copolymerizable photoinitiator; ethyl (2,4,6-trimethylbenzoyl)-phenyl-phosphinate (APO; Omnirad TPOL, IGM Resins, Waalwijk, the Netherlands) was used as a radical photoinitiator; and triethylsilane (TES; Merck, Warsaw, Poland) was used as the telogen. The components were used without additional purification.

### 2.2. Synthesis and Characterization of Syrups

**Figure 1:**
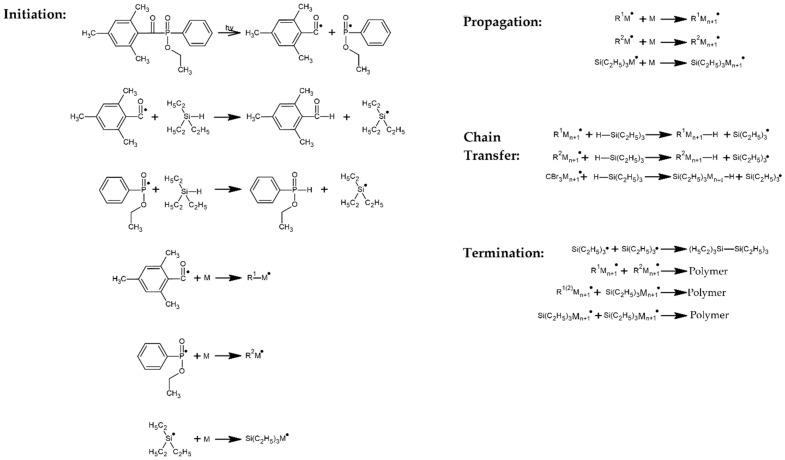
Schematic mechanism of the UV telomerization process (adapted from [17], where M is a taxogen).

The UV telomerization of BA, AA, MMA, and ABP was initiated using the radical photoinitiator APO (0.05, 0.075, and 0.1 wt. parts/100 wt. parts of monomer mixture) and TES (3.5, 7, and 10 wt. parts/100 wt. parts of monomer mixture) as a telogen. As reference samples (R), (met)acrylic syrups were obtained. The reaction mechanism for the cotelomerization is shown in Figure 1.

The UV telomerization processes were realized at 20 °C for 30 min in a glass reactor (250 mL) equipped with a mechanical stirrer and thermocouple under argon as an inert gas. A mixture of monomers (50 g) was introduced into the reactor and purged with argon. A high-intensity UV lamp emitting UV-A radiation (UVAHAND 250, Dr. Hönle AG UV Technology, Gräfelting, Germany) was used as a UV light source. The UV irradiation inside the reactor (15 mW/cm^2^) was controlled with an SL2W UV radiometer (UV-Design, Brachttal, Germany). The compositions of the mixtures and their symbols are presented in Table 1.

The kinetics studies of the UV telomerization process were conducted at 25 °C using the photo-DSC method with a differential scanning calorimeter with UV equipment (Q100, TA Instruments, New Castle, DE, USA). During the measurements, samples of 5 mg were UV-irradiated (320–390 nm) with an intensity of 15 mW/cm^2^ in an argon atmosphere. The polymerization rate (R_p_, %/s) and photoinitiation index (I_p_)—i.e., the ability of the initiation reaction in the tested systems (TES/APO)—were calculated according to Equations (1) and (2), respectively [30]:

(1) $$R_{p}=\frac{\left(\frac{dH}{dt}\right)\;}{H_{0}}\;\left[\%/s\right]$$

(2) $$I_{p}=\frac{R_{p}^{max}}{t_{max}}$$

where *dH*/*dt* is the heat flow recorded during UV irradiation; H_0_ is the theoretical heat value for the complete degree of conversion (∆H = 78.0 kJ/mol for acrylates and ∆H = 54.8 kJ/mol for methacrylates); ∆H_t_ is the reaction heat that has evolved at time t; $R_{p}^{max}$ is the maximum polymerization rate; and $t_{max}$ is the time when the maximum polymerization rate occurs.

The solid content (*SC*) in the syrups was determined using a thermobalance (MA 50/1.X2.IC.A; Radwag, Radom, Poland). Syrups samples (ca. 2 g) were heated in an aluminum pan at 105 °C for 4 h. The *SC* parameter was calculated according to Equation (3):

(3) $$SC=\frac{m_{2}}{m_{1}}\cdot 100\;\left(wt\%\right)$$

where *m*_1_ is the initial weight of a sample and *m*_2_ is the residual weight after the evaporation process.

The dynamic viscosity of the syrups was measured at 25 °C using a DV-II Pro Extra viscometer (spindle #6 or #7, 50 rpm; Brookfield, New York, NY, USA). The K-values for the dry telomers or copolymers were determined using an Ubbelohde viscometer according to the EN ISO 1628-1:1998 standard and the Fikentscher equation (Equation (4)) [31]:

(4) $$K=1000\cdot k=1000\cdot \frac{1.5\log\eta_{r}-1+\sqrt{1+\left(\frac{2}{c}+2+1.5\log\eta_{r}\right)1.5\log\eta_{r}}}{150+300c}$$

where *η_r_* = η/η_0_; η is the viscosity of the telomer/copolymer solution; *η*_0_ is the viscosity of the pure auxiliary diluent (i.e., tetrahydrofurane); and *c* is the telomer/polymer concentration (g/cm^3^).

To determine the presence of TES in the dry telomers, post-reaction mixtures were heated in a vacuum dryer at 60 °C and 10 mm Hg for 1 h. In the spectra of the obtained materials, absorbance was searched for at a wavelength of about 720 cm^−1^ (characteristic for vibrations of Si-C bonds) using FTIR spectroscopy (Nexus FT-IR, Thermo Nicolet, New Castle, DE, USA) [32].

### 2.3. Preparation and Characterization of Self-Crosslinkable Pressure-Sensitive Adhesives

The self-crosslinkable PSAs were composed of only the silicone-(meth)acrylate telomer syrups (i.e., the solutions of the Si telomers in unreacted monomers obtained in the UV telomerization process). As reference samples, PSA films based on (meth)acrylate syrups were used (i.e., the solutions of the (meth)acrylate copolymers in unreacted monomers obtained in the UV photopolymerization process). The syrups were applied onto polyester foils and UV-irradiated (UV irradiation doses were 1, 2, 3, and 4 J/cm^2^) using a medium pressure mercury lamp (UV-ABC; Hönle UV-Technology, Gräfelfing, Germany). The UV exposure was controlled with a radiometer (Dynachem 500; Dynachem Corp., Westville, IL, USA). The base weight of the PSA layers was 60 g/m^2^.

**Figure 2:**
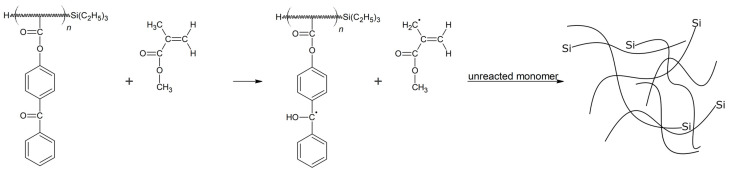
Probable course of photocrosslinking process for Si telomer-based PSAs with pendant benzophenone groups.

The photocrosslinking process in the tested systems took place with the participation of an ABP photoinitiator (hydrogen transfer photoinitiator). This process consists of the production of free radicals through the detachment of the hydrogen atom (from the hydrogen donor molecule) by the triplet ketone (benzophenone group in ABP). When ABP is used, the hydrogen atom is often abstracted from the tertiary carbon atom present in the structure of the comonomers [33]. In this article, we disclose for the first time that the MMA molecule can be the hydrogen donor (such as the case where the hydrogen donor is (CH_3_)_2_C=C (CH_3_)_2_) [34,35]. A proposed course for the photocrosslinking process in the prepared PSAs is shown in Figure 2.

Self-adhesive tests (adhesion to a steel, tack, and cohesion at 20 °C and 70 °C) of the UV-crosslinked PSAs were performed at 23 ± 2 °C and 50% ± 5% relative humidity. The values of adhesion to a steel (also called the peel adhesion) at an angle of 180° were determined according to the AFERA 5001 standard developed by the European Association des Fabricants Europeens de Rubans Auto-Adhesifs (AFERA) using a Zwick/Roell Z010 testing machine (Zwick/Roell, Ulm, Germany). A one-sided PSA film with dimensions of 175 × 25 mm was applied to the degreased steel plate and pressed with a rubber roller weighing 2 kg. The test was performed 20 min after the application of the film to the plate with a peeling speed of 300 mm/min. The tack values were determined with the loop method in accordance with the AFERA 5015 standard using a Zwick/Roell Z010 testing machine (Zwick/Roell, Ulm, Germany). A PSA film with dimensions of 175 × 25 mm was mounted in the upper jaws to obtain loops with the adhesive layer on the outside. The sample was lowered perpendicularly to the degreased steel plate placed in the lower jaws at a speed of 100 mm/min. The contact area was about 6.25 cm^2^. The machine recorded the force needed to detach the adhesive film after brief contact with the steel surface without external forces. The values of cohesion (i.e., the static shear adhesion) were determined in accordance with the AFERA 5012 standard using a device designed by the International Laboratory of Adhesives and Self-Adhesive Materials of the West Pomeranian University of Technology in Szczecin, which enables automatic measurement of the time of joint-crack occurrence. A one-sided adhesive film was applied to the degreased steel plate to form a 25 × 25 mm (6.25 cm^2^) joint and pressed with a 2 kg rubber roller to improve wettability. A 1 kg weight was attached to the free end of the film. The setup was then placed in a tripod so that the force of gravity was exerted on the weld at an angle of 180°. The cohesion value was defined as the time needed for the weld to crack. The test was carried out at temperatures of 20 °C and 70 °C. These parameters were evaluated using three samples for each adhesive film. During self-adhesive properties tests, three types of damage failures may occur: adhesive failure (af), when the adhesive layer remains on the carrier (the cohesion forces are higher than the adhesion forces); cohesive failure (cf), when the adhesive layer remains on both the carrier and the substrate; and mixed failure (mf), when both adhesive and cohesive failures occur. Moreover, the conversion of C=C bonds (DB) in the PSAs after the UV-crosslinking process was analyzed using the FTIR technique (Nexus FT-IR, Thermo Nicolet, New Castle, DE, USA); variations in the absorbance value at 1635 cm^−1^ (C=C bond) and reference value at 1730 cm^−1^ (C=O bond) were monitored according to Equation (5):

(5) $$\mathrm{DB}=\frac{A_{c}^{1635}/A_{c}^{1730}}{A_{u}^{1635}/A_{u}^{1730}}\cdot 100\;\left(\%\right)$$

where $A_{c}^{1635}$ is the absorbance of the crosslinked sample at 1635 cm^−1^; $A_{u}^{1635}$ is the absorbance of the uncrosslinked sample at 1635 cm^−1^; $A_{c}^{1730}$ is the absorbance of the crosslinked sample at 1730 cm^−1^; and $A_{u}^{1730}$ is the absorbance of the uncrosslinked sample at 1730 cm^−1^.

The total conversion of C=C bonds (TC) was determined as the total solids content (assuming that the volatiles constituted 100% of the unreacted monomers in the syrup, as confirmed by comparing the NMR conversion to SC [36]) and the double-bond conversion for the crosslinked films was evaluated according to Equation (6):

(6) TC=((SC/100)+((1−(SC/100))∗(DB/100))·100 (%)

Moreover, the glass-transition temperature (T_g_) values of the UV-crosslinked PSAs were determined with the DSC method (DSC250 differential scanning calorimeter, TA Instruments, New Castle, DE, USA). Samples (ca. 10 mg) were analyzed using hermetic aluminum pans at temperatures from −80 to 200 °C (heating rate of 10 °C/min).

## 3. Results and Discussion

### 3.1. Kinetics of UV-Telomerization Process

**Figure 3:**
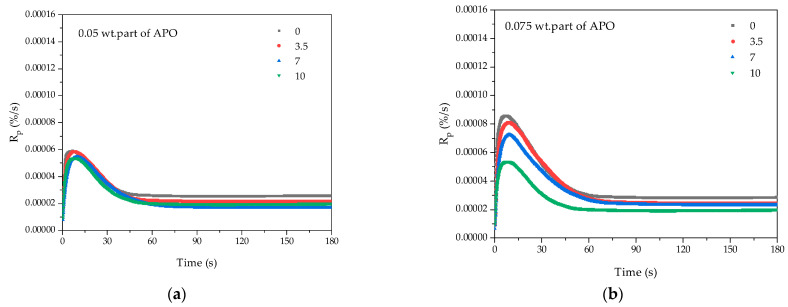

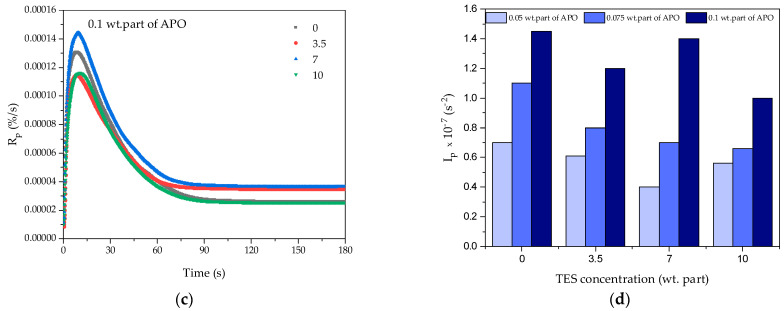
The kinetic curves recorded during UV telomerization of BA, AA, MMA, and ABP in the presence of TES as a telogen (0; 3.5, 7, or 10 wt. parts) initiated by (**a**) 0.05 wt. parts, (**b**) 0.07 wt. parts, and (**c**) 0.1 wt. parts APO and the photoinitiation index of the APO/TES systems (**d**).

First, the influence of the telogen and photoinitiator concentration on the UV-telomerization process in the selected monomer systems was investigated with the photo-DSC method. The results of the kinetic studies for the systems containing 0.05, 0.075, or 0.1 wt. parts APO and 0, 3.5, 7, or 10 wt. parts TES are presented in Figure 3.

Kinetic studies revealed that the reaction rate was strongly dependent on the APO and TES concentrations. As can be seen (Figure 3a–c), the higher the APO concentration in the reaction mixture was, the faster the reaction rate. At a low concentration of APO, there were no significant differences in the effects of TES on the rate of reaction (Figure 3a). With an average amount of APO, more TES in the system led to a slower reaction, and the reaction was fastest without telogen (Figure 3b). Interestingly, there was no increasing or decreasing tendency in the influence of the TES concentration on the reaction rate in the case of mixtures with 0.1 wt. parts APO. In these systems, the highest R_p_ was recorded with 7 wt. parts TES, and the smallest R_p_ values for 3.5 wt. parts TES. However, the highest concentration of TES tended to slow down the reaction rate (Figure 3a,d). Moreover, the kinetic studies highlighted a characteristic feature of the telomerization process with TES; namely, that after 60 s of irradiation, there was a rapid decrease in the reaction rate. However, in systems with medium TES content (3.5 or 7 wt. parts) and 0.1 wt. parts APO, the process ran further but only at a low rate (Figure 3c). Regarding the abilities of APO and TES to initiate the process, it can be seen (Figure 3d) that the more photoinitiator there was, the higher the Ip value (regardless of the TES concentration). Moreover, the highest Ip values were found in the arrangement without TES. With the increase in telogen concentration, the initiating abilities of the APO/TES system decreased. One exception was the mixture with 0.1 wt. parts APO/7 wt. parts TES (similar Ip values as for 0.1 wt. parts APO without TES).

### 3.2. The Physicochemical Properties of Syrups

**Figure 4:**
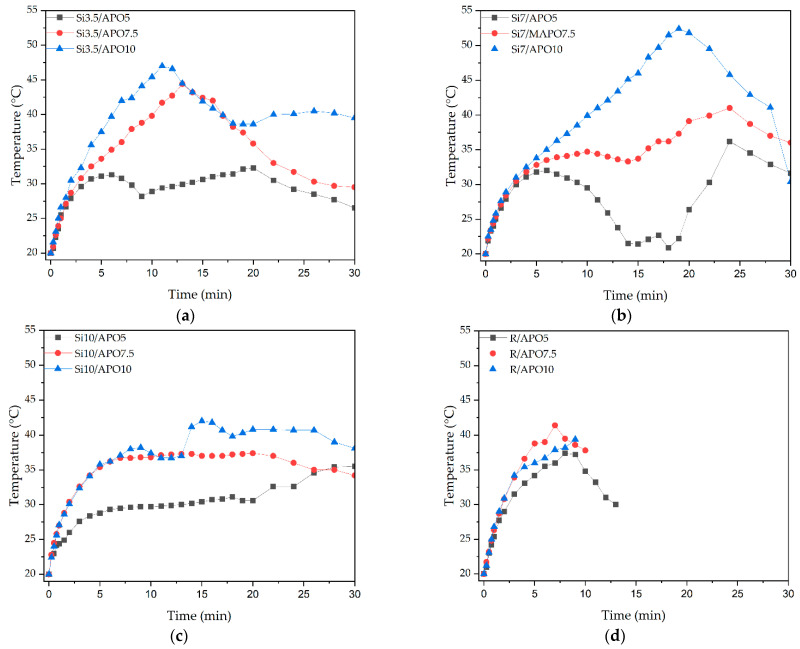
The temperature change in the reactor during the UV-telomerization process for 3.5 (**a**), 7 (**b**), or 10 (**c**) wt. parts TES and during the photopolymerization process (**d**).

The courses of the UV-telomerization process (with TES) and UV-photopolymerization (reference samples without TES) in the glass reactor (with the desired mixing speed for the reactants) were investigated by recording the time dependences of the mixture temperature; thermographs for the systems with different contents of APO and TES are presented in Figure 4.

The presented thermograms revealed, first of all, that the UV-telomerization process could be carried out for a longer duration (up to 30 min) than the photopolymerization process with the same APO content (up to 9–14 min). The shorter reaction time for the reference syrups (R, Figure 4d) was caused by the winding up of the reaction products on the stirrer (gel formation), which did not occur for the telomer syrups. The maximum temperature values for photopolymerization were 37–42 °C. However, the values of the temperature peaks in the telomeric systems were generally higher (reaching 53 °C for Si7/APO-10), which was in agreement with the photo-DSC results (for this system, the reaction rate and Ip values were the highest; see Figure 3c,d). The presented thermographs also confirmed that, with the increase in the APO content in the telomeric mixtures, the recorded peak temperature became higher and occurred faster. An interesting observation was the occurrence of a double (Si3.5/APO-10; Si7/APO-7.5) or even triple temperature peak (Si10/APO-10), which in some arrangements was unobservable (Si7/APO-10; probably due to the high first peak) and may indicate a process with two or more stages. This was confirmed by the kinetic studies results, which did not show a simple correlation between TES concentration and the course of the telomerization reaction. Additionally, the presented thermograms revealed a multistage course for the reaction. It should be emphasized that this was also influenced by the mechanical mixing of the reactants during irradiation. Nevertheless, the UV-telomerization process allowed the production of liquid syrups.

**Figure 5:**
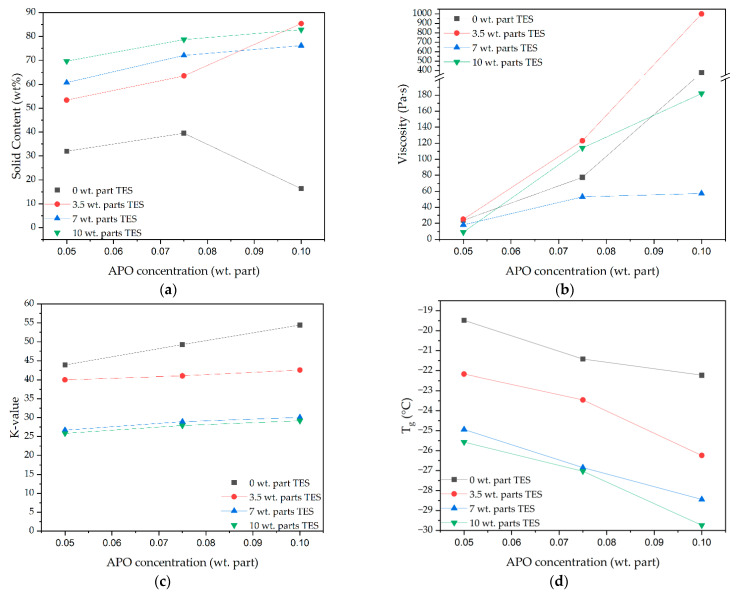
Solid content (**a**) and dynamic viscosity (**b**) of syrups and K-values (**c**) and T_g_ (**d**) of silicone-(meth)acrylate telomers and (meth)acrylate copolymers.

The results for the solid content and dynamic viscosity of the obtained syrups, as well as the K-values and glass-transition temperatures (T_g_) of the dry silicone-(meth)acylate telomers and (meth)acrylate copolymers, prepared with different APO and TES concentrations are presented in Figure 5.

As can be seen, the UV-telomerization products (i.e., the silicone-(meth)acrylate telomer solutions) were characterized by higher SC values than the photopolymerization process products (i.e., the (meth)acrylate copolymer solutions); specifically, 53–87% and <40%, respectively. Additionally, as the contents of APO and TES increased, the SC values increased. A decrease in the SC value was recorded only for the R/APO-10 sample (18%). It was mentioned earlier that, during the photopolymerization, the viscosity of the reaction mixture increased very quickly (the gel effect), which indirectly indicated the high molecular weights of the products. However, the SC values were low for (meth)acrylate syrups, so the monomer conversion during photopolymerization was also lower than during telomerization. This result for photopolymerization was expected, as the overall reaction rate of this process was higher than that of the telomerization process, resulting in a gel effect and low monomer conversion. The high molecular weights of the photopolymerization products were confirmed by their greater K-values, ranging from 45 to 55 a.u. (Figure 5c).

The influence of the concentrations of APO and TES on the dynamic viscosity values of the syrups was determined. Only when the APO concentration was increased did the dynamic viscosity values increase. However, there was no correlation with the TES concentration in the system. The lowest APO concentration (0.05 wt. part) led to low-viscosity liquid-process products (10–30 Pa·s) suitable for coating carriers in the production of PSAs. In addition, for the same systems, it was observed that more telogen resulted in lower dynamic viscosity in the telomer syrups. The highest values for the dynamic viscosity (>900 Pa·s) were revealed for Si3.5/APO-10 syrups. Unexpectedly, the Si7 syrups showed much lower dynamic viscosity than the others (20 Pa·s for Si7/APO-5 and ca. 50 Pa·s for Si7/APO-7.5 and Si7/APO-10). In these systems, the telomerization process also took place differently from the others, which was confirmed by the thermograms (Figure 4b). Nevertheless, it should be mentioned that the obtained dynamic viscosity values were influenced by both the telomer/oligomer content (or copolymers, as in the case of the reference samples) and their molecular weights, as well as the amounts of unreacted monomers in the syrups. The determined K-values were indirectly indicative of the molecular weight values of the obtained oligomers and copolymers (Figure 5c). Generally, higher K-values were observed for copolymers than for telomeres, as expected. Additionally, with increasing APO concentrations, K-values increased. However, with more telogen in the system, lower K-values were obtained, and the results for Si7 and Si10 syrups were similar (26–28 a.u.).

The new silicone-(meth)acrylate telomers obtained were also characterized in terms of their glass-transition temperature (Figure 5d). The Si telomers exhibited significantly lower T_g_ values (−22 °C for Si3.5/APO-5 and −29.5 °C for Si10/APO-10) than those for the reference copolymers (−19.5 °C for R/APO-5 and −21.5 °C for R/APO-10). The T_g_ values decreased with increases in both TES and APO concentrations. Thus, telomeres with lower molecular weights (lower K-values) and higher amounts of silicon atoms in the structure were characterized by lower T_g_. This type of telomer could have a positive effect on the applicability of the prepared PSAs.

**Figure 6:**
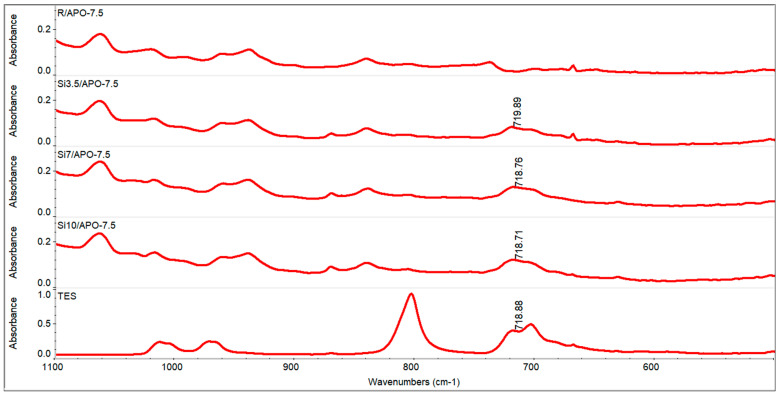
FT-IR spectra of TES, dry systems consisting of Si telomers (with the same content of 0.075 wt. parts APO), and dry reference with the same APO concentration.

To confirm the incorporation of triethylsilane into the oligomeric chains, FTIR analysis of the dry telomers and reference copolymer was performed. For this purpose, unreacted monomers were evaporated from the syrups by heating them in a vacuum dryer (at 60 °C and 10 mm Hg) for 1 h. The IR absorption band corresponding to the stretching vibration of the Si-C bond and located around 720 cm^−1^ [32] was detected both for pure TES and the synthesized telomers. However, it was not detected for the reference sample. This confirmed the incorporation of the TES moiety into the oligomeric chains of the Si telomers. The FT-IR spectra are shown in Figure 6.

Due to the high solids content (>60%) and low glass-transition temperature, as well as their appropriate viscosity, the syrups based on 0.075 wt. parts APO (i.e., Si3.5/APO-7.5, Si7/APO-7.5 and Si10/APO-7.5) could be used to produce self-crosslinkable pressure-sensitive adhesives.

### 3.3. Properties of UV-Crosslinked PSA

**Figure 7:**
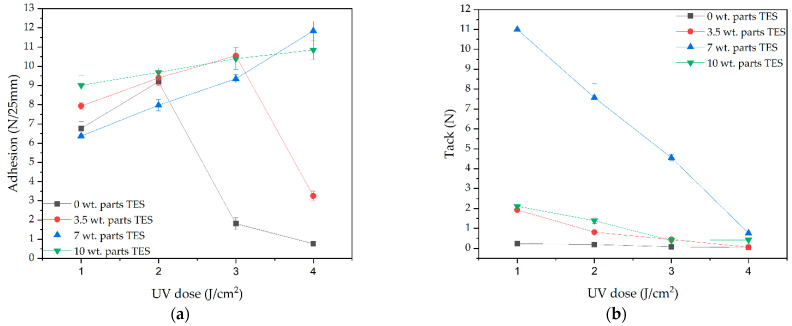

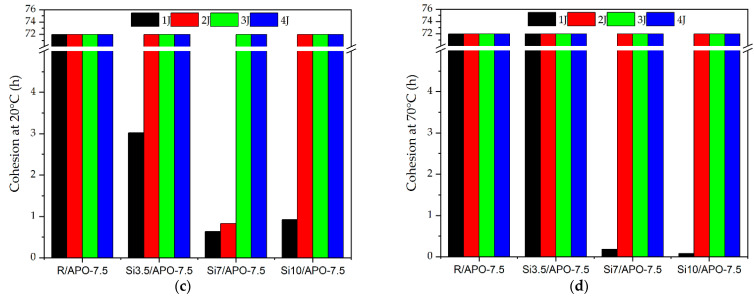
Self-adhesive properties of PSAs based on Si telomere syrups with the same amounts of APO (0.075 wt. parts): (**a**) adhesion to steel, (**b**) tack, and (**c**) cohesion at 20 °C and (**d**) 70 °C.

The self-adhesives properties of PSA films based on silicone-(meth)acrylate syrups or (meth)acrylate copolymer syrups after the UV-crosslinking process (with UV energy doses of 1, 2, 3, or 4 J/cm^2^) are presented in Figure 7. As is known, the crosslinking process is critical in the preparation of self-adhesive materials and can provide them with the desired peculiarities; in particular, cohesion. Moreover, as can be seen in Figure 2, the proposed mechanism for crosslinking both in telomeric and copolymeric syrups involves abstracting a hydrogen atom from an unreacted MMA molecule using a carbonyl moiety of an ABP unit to generate reactive radicals able to further chemically combine with the unreacted monomer in the system, with the final result of telomer/copolymer branching and then crosslinking. The results for the self-adhesive properties, which indirectly prove the correctness of the process, are presented in Figure 7.

Thus, the values for the adhesion to a steel surface for the PSAs without Si atoms (i.e., PSAs based on the R/APO-7.5 syrup) were relatively low in comparison with the PSAs based on Si telomers. The highest value (9 N/25 mm) was recorded for the sample subjected to 2 J/cm^2^ UV-dose irradiation. In the case of the PSA systems based on the Si telomer syrups, the maximum value for adhesion was found to be 12.4 N/25 mm for Si7/APO-7.5 at a UV dose of 4 J/cm^2^. However, it should be noted that the adhesion values depended on the UV dose (the higher the UV dose, the higher the adhesion). It is known that a higher UV dose promotes cleavage of the π bonds of the C=C bonds belonging to the unreacted monomer molecules and the formation of a denser polymer network. In the case of the PSAs based on R/APO-7.5, the lowest adhesion values were recorded after irradiation with 3 and 4 J/cm^2^ (1.5 and 0.8 N/25 mm, respectively). As shown above, this system contained (meth)acrylate copolymer (40% SC) with a relatively high molecular weight (high K-value) and a large amount of unreacted monomers (60%). Thus, at the irradiation stage, a dense polymer network was formed (more monomers were involved in its formation than in the telomer systems). Hence, the stiffness of the formed polymer network limited its adhesion to the steel substrate. In the case of PSAs based on telomeric syrups, generally higher adhesion values were found for those with the highest proportions of Si atoms and the smallest molecular weights (lowest K-values); i.e., Si10/APO-7.5 (9–11 N/25 mm). On the other hand, a significant decrease in adhesion (down to 3 N/25 mm) was noticed for PSAs based on Si3.5/APO-7.5 at the maximum UV-dose irradiation, and a more considerable proportion of telomers and higher molecular weights (SC ca. 60%, K-value ca. 40 a.u.) were observed than for the other telomeric systems. The tack results for the obtained PSAs were interesting. Generally, the tack values decreased with increasing UV dose due to the increase in the crosslinking density of the systems, which has been described previously in the literature [11,37]. However, only the PSAs from the Si7/APO-7.5 syrup showed high tack values (11.5 N after 1 J/cm^2^ UV dose). All the other systems displayed lower tack values below 2.5 N, with the lowest value recorded for the reference sample (<0.5 N).

The cohesion at both 20 °C and 70 °C for most of the PSAs reached very high values (above 72 h). However, this value decreased when the TES and APO concentrations increased. This was due to the decreasing K-value (lower molecular weights), as well as the production of increasingly stiffer films, which can exhibit wetting problems, as indicated by the presence of adhesion failure when tested at 20 °C. Excellent cohesion at 70 °C also supports this finding, as the wettability of the adhesive films at elevated temperature was higher. During the tests of the self-adhesive properties, only adhesive cracks were observed. Due to the very good self-adhesive properties found for most of the compositions, only samples crosslinked with the dose of 3 J/cm^2^ were further investigated.

Double-bond conversions in the crosslinked adhesive films determined with FT-IR, SC, and total conversion are shown in Table 2.

The conversion of unreacted monomers during the UV crosslinking of PSAs (DB) was closely related to the amounts of unreacted monomers in the telomeric syrups from which the adhesive films were obtained. As can be seen, the highest DB value (84%) was recorded for R/APO-7.5 (reference sample), but this was due to the large amount of unreacted monomers in the starting syrup (60%) and the low linear (meth)acrylate copolymer content, which facilitated the migration of the radicals formed. In the case of the telomeric systems, the DB values were lower (76–79%). Considering the total conversion of the C=C bonds in the PSAs after the UV-crosslinking process, the highest value was recorded for Si10/APO-7.5 (96%). It should be noted that the conversion of unreacted monomers—and, thus, the UV-photocrosslinking process—was most effective in the system with the highest silicon moiety content (10 wt. parts). This was due to the system having the lowest molecular weights for the Si10 telomers containing benzophenone groups (from ABP) in their side chains (the lowest K-values), which made the mobility of such macroradicals higher and the photocrosslinking process more effective.

**Figure 8:**
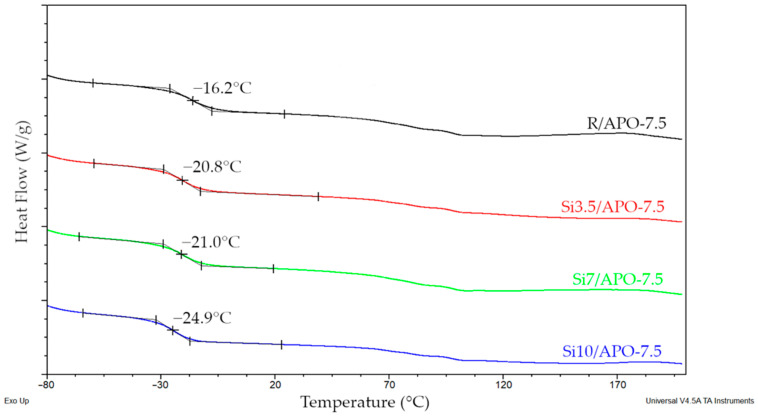
DSC thermograms of the selected PSAs crosslinked under UV radiation with a dose of 3 J/cm^2^.

Eventually, the values of the glass-transition temperatures measured for the UV-crosslinked PSAs based on Si telomers and (meth)acrylate copolymers (Figure 8) reached accordance with the content of the TES moiety in the final telomer products.

As can be seen, PSAs based on Si telomers were characterized by significantly lower T_g_ values compared to the reference sample (a difference of almost 9 °C). This was because of the incorporation of TES molecules into the chain; the more TES residues in the system, the higher the chain flexibility and, consequently, the lower the T_g_. This result is very satisfactory for self-adhesive materials.

## 4. Conclusions

Silicone-(meth)acrylic telomers with terminal Si atoms were prepared via a UV-telomerization process using triethylsilane (TES) as a telogen. The prepared Si telomer syrups were used as adhesive binders to obtain self-crosslinkable pressure-sensitive adhesives without any additional crosslinking agent. The main conclusions are as follows:

- The UV telomerization of the tested (meth)acrylate monomers was strongly dependent on the radical photoinitiator concentration, and there was no trend observed in the TES concentration; only at the average APO content was a decrease in the reaction rate found with an increase in the concentration of telogen;
- Silicone-(meth)acrylate telomer solutions were characterized by higher solid content values (>60%) and lower dynamic viscosity than the reference (meth)acrylate syrups;
- Si telomers exhibited significantly lower K-values and T_g_ values (−29.5 °C) than the (meth)acrylate copolymers (−21.5 °C);
- Si telomer-based self-crosslinking PSAs were distinguished by excellent adhesion to steel (up to 12.4 N/25 mm) and tack (11 N) and cohesion both at 20 °C and 70 °C (>72 h); in addition, they demonstrated low T_g_ compared to the reference sample (difference of up to almost 9 °C).

The results of the studies carried out and described here encourage the authors to work further on the preparation of new Si telomeres (with other silicon telogens) and analyze their structures and the thermo-mechanical properties of the PSAs obtained from them.

## Figures and Tables

**Table 1:** Compositions of the monomers, photoinitiator, and telogen for the preparation of silicone-(meth)acrylic telomer syrups (Si) and (meth)acrylic syrups (R).

Syrups Acronym	Monomers (wt.%)				APO (wt. part) *	TES (wt. parts) *
	BA	AA	MMA	ABP		
R/APO-5	86.5	7.5	5	1	0.05	0
R/APO-7.5					0.075	0
R/APO-10					0.1	0
Si3.5/APO-5					0.05	3.5
Si3.5/APO-7.5					0.075	3.5
Si3.5/APO-10					0.1	3.5
Si7/APO-5					0.05	7
Si7/APO-7.5					0.075	7
Si7/APO-10					0.1	7
Si10/APO-5					0.05	10
Si10/APO-7.5					0.075	10
Si10/APO-10					0.1	10

* per 100 wt. parts of monomer mixtures.

**Table 2:** Double-bond conversion determined with FT-IR spectroscopy and total monomer conversion.

PSA	Content of Unreacted Monomers in the Adhesive Film before UV Crosslinking ^1^	Conversion of C=C Bonds in PSAs during UV-Crosslinking Process (DB)	Total Conversion of C=C Bonds (TC)
	(%)	(%)	(%)
R/APO-7.5	60	84	90
Si3.5/APO-7.5	36	75	91
Si7/APO0-7.5	28	72	92
Si10/APO7.5	21	81	96

^1^—Based on SC calculation.

## Data Availability

The data presented in this study are available on request from the corresponding author.

## References

[1] Benedek I., Feldstein M.M. (2008). Fundamentals of Pressure Sensitivity.

[2] Park H.W., Park J.W., Lee J.H., Kim H.J., Shin S. (2019). Property Modification of a Silicone Acrylic Pressure-Sensitive Adhesive with Oligomeric Silicone Urethane Methacrylate. Eur. Polym. J..

[3] Seok W.C., Park J.H., Song H.J. (2022). Effect of Silane Acrylate on the Surface Properties, Adhesive Performance, and Rheological Behavior of Acrylic Pressure Sensitive Adhesives for Flexible Displays. J. Ind. Eng. Chem..

[4] Dastjerdi Z., Cranston E.D., Dubé M.A. (2018). Pressure Sensitive Adhesive Property Modification Using Cellulose Nanocrystals. Int. J. Adhes. Adhes..

[5] Lee S.W., Park J.W., Lee Y.H., Kim H.J., Rafailovich M., Sokolov J. (2012). Adhesion Performance and UV-Curing Behaviors of Interpenetrated Structured Pressure Sensitive Adhesives with 3-MPTS for Si-Wafer Dicing Process. J. Adhes. Sci. Technol..

[6] White C.C., Tan K., Wolf A.T., Carbary L.D. (2010). Advances in Structural Silicone Adhesives.

[7] Lin S.B., Durfee L.D., Ekeland R.A., McVie J., Schalau G.K. (2007). Recent Advances in Silicone Pressure-Sensitive Adhesives. J. Adhes. Sci. Technol..

[8] Cheng J., Li M., Cao Y., Gao Y., Liu J., Sun F. (2015). Synthesis and Properties of Photopolymerizable Bifunctional Polyether-Modified Polysiloxane Polyurethane Acrylate Prepolymer. J. Adhes. Sci. Technol..

[9] Balaban M., Antić V., Pergal M., Godjevac D., Francolini I., Martinelli A., Rogan J., Djonlagić J. (2013). Influence of the Chemical Structure of Poly(Urea-Urethane-Siloxane)s on Their Morphological, Surface and Thermal Properties. Polym. Bull..

[10] Kostyuk A., Ignatenko V., Smirnova N., Brantseva T., Ilyin S., Antonov S. (2014). Rheology and Adhesive Properties of Filled PIB-Based Pressure-Sensitive Adhesives. I. Rheology and Shear Resistance. J. Adhes. Sci. Technol..

[11] Benedek I., Feldstein M.M. (2019). Technology Pressure-Sensitive Adhesives and Products.

[12] Satas D. (1989). Handbook of Pressure Sensitive Adhesive Technology.

[13] Czech Z., Kowalczyk A., Górka K., Głuch U., Shao L., Świderska J. (2012). Influence of the Unsaturated Photoinitiators Kind on the Properties of Uv-Crosslinkable Acrylic Pressure-Sensitive Adhesives. Pol. J. Chem. Technol..

[14] Bolshakov A.I., Kuzina S.I., Kiryukhin D.P. (2017). Tetrafluoroethylene Telomerization Initiated by Benzoyl Peroxide. Russ. J. Phys. Chem. A.

[15] Chen J., Chalamet Y., Taha M. (2003). Telomerization of Butyl Methacrylate and 1-Octadecanethiol by Reactive Extrusion. Macromol. Mater. Eng..

[16] Starks C.M. (1974). Free Radical Telomerization.

[17] Boutevin B. (2000). From Telomerization to Living Radical Polymerization. J. Polym. Sci. A Polym. Chem..

[18] Loubat C., Boutevin B. (2001). Telomerization of Acrylic Acid with Mercaptans: Part 2. Kinetics of the Synthesis of Star-Shaped Macromolecules of Acrylic Acid. Polym. Int..

[19] Loubat C., Boutevin B. (2000). Telomerization of Acrylic Acid with Thioglycolic AcidEffect of the Solvent on the CT Value. Polym. Bull..

[20] Boyer C., Boutevin G., Robin J., Boutevin B. (2007). Synthesis of Macromonomers of Acrylic Acid by Telomerization: Application to the Synthesis of Polystyrene-g-poly (Acrylic Acid) Copolymers. J. Polym. Sci. A Polym. Chem..

[21] Li G.H., Yang P.P., Zhao Z.J., Gao Z.S., He L.Q., Tong Z.F. (2011). Synthesis of Poly (Tert-Butyl Acrylate) Telomer by Free-Radical Telomerization. CIESC J..

[22] Jeanmaire T., Brondino C., Hervaud Y., Boutevin B. (2002). Synthesis of new phosphonic derivatives bearing a perfluorinated chain and their adhesive properties on steel. Phosphorus Sulfur Silicon Relat. Elem..

[23] Tan B., Pan H., Irshad H., Chen X. (2002). Synthesis of Ultraviolet-Curable Modified Polysiloxane and Its Surface Properties. J. Appl. Polym. Sci..

[24] Weisbrodt M., Kowalczyk A., Kowalczyk K. (2021). Structural Adhesives Tapes Based on a Solid Epoxy Resin and Multifunctional Acrylic Telomers. Polymers.

[25] Ghosh P., Mitra P.S. (1977). Novel Solvent Effects in the Photopolymerization of Methyl Methacrylate by Use of Quinoline–Bromine Charge-transfer Complex as Photoinitiator. J. Polym. Sci. Polym. Chem. Ed..

[26] Ghosh P., Banerjee H. (1973). Photopolymerization of Methyl Methacrylate With the Use of Bromine As Photoinitiator. J. Polym. Sci. Polym. Chem. Ed..

[27] Kowalczyk A., Weisbrodt M., Schmidt B., Kraśkiewicz A. (2021). The Effect of Type-I Photoinitiators on the Kinetics of the UV-Induced Cotelomerization Process of Acrylate Monomers and Properties of Obtained Pressure-Sensitive Adhesives. Materials.

[28] Kowalczyk A., Weisbrodt M., Schmidt B., Gziut K. (2020). Influence of Acrylic Acid on Kinetics of UV-Induced Cotelomerization Process and Properties of Obtained Pressure-Sensitive Adhesives. Materials.

[29] Spitzer J.J., Midgley A., Takamura K., Lok K.P., Xanthopoulo V.G., Ditrich U. (1994). Novel Polytelomerization Process and Polytelomeric Products. WO Patent.

[30] Kabatc J. (2017). The Influence of a Radical Structure on the Kinetics of Photopolymerization. J. Polym. Sci. A Polym. Chem..

[31] Coelho J.F.J., Gonçalves P.M.F.O., Miranda D., Gil M.H. (2006). Characterization of Suspension Poly(Vinyl Chloride) Resins and Narrow Polystyrene Standards by Size Exclusion Chromatography with Multiple Detectors: Online Right Angle Laser-Light Scattering and Differential Viscometric Detectors. Eur. Polym. J..

[32] Omar M.F., Ismail A.K., Sumpono I., Alim E.A., Nawi M.N., Rahim Mukri M.‘A., Othaman Z., Sakrani S. (2012). FTIR Spectroscopy Characterization of Si-C Bonding in SiC Thin Film Prepared at Room Temperature by Conventional 13.56 MHz RF PECVD. Malays. J. Fundam. Appl. Sci..

[33] Czech Z., Loclair H. (2005). Investigations of UV-Crosslinkable Water-Soluble Acrylic Pressure-Sensitive Adhesives. Polimery.

[34] Turro N.J. (1978). Modern Molecular Photochemistry.

[35] Pączkowski J. (2003). Fotochemia Polimerów: Teoria i Zastosowanie.

[36] Gziut K., Kowalczyk A., Schmidt B. (2020). Free-Radical Bulk-Photopolymerization Process as a Method of Obtaining Thermally Curable Structural Self-Adhesive Tapes and Effect of Used Type I Photoinitiators. Polymers.

[37] Czech Z., Kowalczyk A., Kabatc J., Świderska J. (2012). UV-Crosslinkable Acrylic Pressure-Sensitive Adhesives for Industrial Application. Polym. Bull..

